# Label-free toxicology screening of primary human mesenchymal cells and iPS-derived neurons

**DOI:** 10.1371/journal.pone.0201671

**Published:** 2018-09-04

**Authors:** Maria Serena Piccinno, Tiziana Petrachi, Elisa Resca, Valentina Strusi, Valentina Bergamini, Giuseppe Antonio Mulas, Giorgio Mari, Massimo Dominici, Elena Veronesi

**Affiliations:** 1 Science & Technology Park for Medicine (TPM), Mirandola, Italy; 2 Department of Medical and Surgical Sciences for Children & Adults, University-Hospital of Modena and Reggio Emilia, Modena, Italy; Newcastle University, UNITED KINGDOM

## Abstract

The high-throughput, label-free Corning Epic assay has applications in drug discovery, pharmacogenomics, cell receptor signaling, cell migration, and viral titration. The utility of Epic technology for biocompatibility testing has not been well established. In manufacturing of medical devices, in vitro and in vivo biocompatibility assessments are mandatory, according to ISO 10993. The new medical device regulation MDR 745/2017 specifies that ex vivo assays that can closely recapitulate in vivo scenarios are needed to better evaluate biomedical devices. We propose herein that Epic technology—which enables detection of variations in cell mass distribution—is suitable for biocompatibility screening of compounds. In this study, we challenged primary human osteoblasts, endothelial cells, and neurons derived from induced pluripotent stem cells with specific concentrations of methyl methacrylate (MMA). Polymeric MMA has long been applied in cranioplasty, where it makes contact with multiple cell types. Application of Epic technology yielded real-time cytotoxicity profiles for all considered cell types. The results were compared with those from microscopic observation of the same culture plate used in the Epic analyses. The Epic assay should be further examined for its utility for cell biology, genomics, and proteomics companion assays. Our results suggest that Epic technology can be applied to biocompatibility evaluation of human cells in medical device development.

## Introduction

Medical devices in development must be evaluated for biocompatibility in accordance with ISO 10993 [1]. This includes cytotoxicity and in vivo tests, such as irritation, intracutaneous reactivity, and sensitization, for all classes of medical devices [2,3,4]. To ascertain cytotoxicity, ISO 10993–5 mandates use of the methylthiazolyl tetrazolium (MTT) assay with 3T3 or L929 murine cells cultured with extracts derived from test samples, obtained according to ISO 10993–12 [5]. In the MTT assay, tetrazolium salt is added to cells in culture; the salt is reduced by viable cells into a colored formazan, which is quantifiable by a colorimetric measurement [6]. However, the MTT assay has several limitations, the most important being that tetrazolium is toxic to cells. Therefore, the MTT assay must be undertaken with separate cell culture plates for each time point [7,8,9]. This assay also is associated with a long incubation time (up to 4 hours) prior to colorimetric detection. Thus, the initial cell response cannot be observed with this method [6].

**Fig 1:**
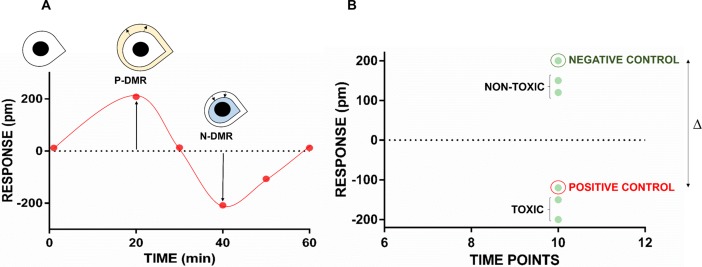
Cell phenotypic profile results of the Epic label-free assay, and implications for toxicology testing. Fig **1A**: After administration of the compound, a positive-dynamic mass redistribution (P-DMR) indicated an increase in the cell response and correlated with augmented cell size. Conversely, a decrease in the cell response was observed as a negative-dynamic mass redistribution (N-DMR) and correlated with cell shrinkage. Fig **1B**: The difference in response (Δ) between the negative (green circle) and positive controls (red circle) enabled discrimination of nontoxic compounds (i.e., similar to the negative control) from toxic compounds (i.e., similar to the positive control).

Epic label-free technology (Corning, Tewksbury, MA) has been described as a means of cell phenotypic screening in drug discovery [10,11] and may be suitable for analyses of biocompatibility. The Epic assay is noninvasive and is performed by placing native cells onto optical biosensors embedded in each well of a microplate [12]. The assay results specify the dynamic mass redistribution (DMR)—that is, variations in the steric distribution of cell mass—in picometers (pm) [13]. An increase in raw signal (response) is correlated with a volumetric increase and is denoted as a positive-DMR (P-DMR). Conversely, a decrease in response is associated with cell shrinkage and is reported as a negative-DMR (N-DMR; Fig 1) [14,15]. The temporal sequence of all DMR phases generates a phenotypic profile for the corresponding cell population.

In toxicologic studies involving the Epic assay, shrinkage associated with apoptosis yields an N-DMR profile, which indicates toxicity (Fig 1A) [16]. Cell swelling, associated with cell necrosis, may produce assay results of a P-DMR followed by an N-DMR, with the latter indicating cell membrane degradation [17]. Appropriate controls are included in an Epic analysis at specific time points to discriminate nontoxic from toxic compounds. The former produces a response similar to that of the negative control, whereas the latter generates results similar to that of the positive control (Fig 1B).

Epic label-free technology can be applied to diverse fields, including agonist-antagonist assays, identification of cytotoxic agents, and drug development in pharmacology [18,19].

The use of the Epic assay to evaluate biocompatibility of medical devices has not been explored previously. Pikis et al. evaluated a series of 6 patients who experienced neuronal dysfunction after polymethyl methacrylate (PMMA) cranioplasty; these authors noted that neurotoxicity could be related to the release of the methyl methacrylate (MMA) monomer during implantation of the cranial prosthesis [20]. Herein, we applied Epic technology to ascertain the biocompatibility of MMA in multiple cell types: primary human adherent endothelial cells, osteoblast lines, and neurons derived from induced pluripotent stem cells (i.e., iPS-derived neurons). Our results demonstrate that biocompatibility analyses of medical devices are feasible with the Epic assay. In our hands, Epic label-free technology was sufficiently sensitive to potentially allow for customized assays of human cells.

## Materials and methods

### Cell cultures

Primary human osteoblasts and HUVECs, as mesenchymal cells, were used to represent bone and blood vessels, respectively. Primary human osteoblasts and all cell culture reagents were purchased from PromoCell (Heidelberg, Germany). HUVECs were purchased from PromoCell, and all cell culture reagents were obtained from Gibco (Life Technologies, Paisley, UK). For neuronal tissue, we used iPS-derived neurons (iCell i-Neurons; Cellular Dynamics International, Madison, WI).

Primary human osteoblasts were thawed and subcultured, per the manufacturer’s instructions. Cells were incubated for 2 minutes in a water bath at 37°C, and a suspension of approximately 500,000 cells/vial was transferred to a cell culture flask at a density of 20,000 cells/cm^2^. The flask had been prefilled with growth medium (PromoCell) and kept in an incubator at 37°C and 5% CO_2_. The medium was replaced 24 hours after thawing and every 3 days thereafter. At 90% confluency, osteoblasts were detached with a solution of trypsin and Ethylenediaminetetraacetic acid (EDTA) (DetachKit, PromoCell) for 3 minutes at room temperature. Fetal bovine serum (FBS) was added to inactivate the trypsin, and cells were seeded in media at 10,000 cells/cm^2^. At passage 7, cells were detached and seeded at 6000 cells/well into a label-free 96-well microplate (PerkinElmer, Waltham, MA) for cytotoxicity assays.

After thawing, 300,000 HUVECs were resuspended in M200 basal medium supplemented with an LSGS (i.e., low serum growth supplement) kit comprising 2% FBS, 3 ng/mL of rh-bFGF (i.e., recombinant human basic fibroblast growth factor), 10 μg/mL of heparin, 100 μg/mL of bovine serum albumin (BSA), 1 μg/mL of hydrocortisone, 10 ng/mL of rh-EGF (i.e., recombinant human epidermal growth factor), 10 μg/mL of gentamicin, and 0.25 μg/mL of amphotericin B. Cells were seeded into flasks precoated with an attachment factor (AF, purchased by Gibco) at a density of 4000 cells/cm^2^. The AF was added to the flask and kept at 37°C for 30 minutes, than it was discarded and the flask was washed with PBS 1x. The medium was replaced 24 hours after thawing and every 3 days thereafter. At 90% confluency, HUVECs were detached by treatment with trypsin/EDTA for 5 minutes at room temperature. FBS was added for trypsin inactivation and washing, and cells were seeded into precoated flasks at a density of 4000 cells/cm^2^. Cells then were amplified for several passages. At passage 7, cells were detached and seeded at a density of 9000 cells/well into a label-free 96-well microplate (PerkinElmer) for cytotoxicity assays.

For cytotoxicity assays involving human iPS-derived neurons, label-free 96-well sensor microplates (PerkinElmer) were coated with a base layer of poly-L-ornithine and an upper layer of laminin (both, Sigma, Milan, Italy). Complete Maintenance Medium (Cellular Dynamics International) then was added to wells, and iPS-derived neurons were thawed and immediately seeded at a density of 30,000 cells/well.

### Label-free cytotoxicity assay

In cytotoxicity studies with the Epic assay, the negative control (nontoxic compound) was 0.2% DMSO, and the positive control (toxic compound) was 0.1% SDS. Seven time points were considered: 1 minute, 20 minutes, and 1, 2, 4, 8, and 24 hours. Concurrently, continuous-mode readings were taken for all cell types at a frequency of 1 read every 5 minutes to investigate the early-stage phenotypic profile (i.e., within the first hour).

Cells were seeded in single wells containing 120 μL of specific culture media, and MMA (Tecres SpA, Verona, Italy) was added at a concentration of 250, 550, or 700 μg/cm^2^. Twenty-four hours after seeding, cells were washed 4 times using 49 μL of an assay buffer prepared from a cell-specific complete culture medium diluted 1:1 with double-distilled water. Washing was carried out using an aspiration wand (VP 185-L, V&P Scientific, San Diego, CA), and a final volume of 80 μl/well of assay buffer was reached. The loaded microplates were covered with a permeable film membrane (SealMate AeraSeal; Excel Scientific, St. Louis, MO) and were equilibrated at 37°C and 5% CO_2_ for 2 hours. After incubation, an initial baseline reading was taken for 5 minutes, and 20 μL/well of each tested compound (at 5× concentration) was transferred to cell-loaded label-free microplates as follows: (1) assay buffer (blank sample), (2) 0.1% SDS in assay buffer (v/v, final concentration; positive cytotoxic control), (3) 0.2% DMSO in assay buffer (v/v, final concentration; negative nontoxic control), and (4) MMA at the prespecified concentrations in assay buffer.

Immediately after compound addition, the plates were gently mixed with an IncuShaker Mini (Memmert, Schwabach, Germany) at 75 rpm for 30 seconds. Plates then were loaded into the Enspire Multimode Plate Reader (PerkinElmer), and final readings were taken sequentially at 5 time points: (1) 1 to 60 minutes (1 read repeat/minute), (2) 1.50 to 2 hours (1 read repeat/minute), (3) 3.50 to 4 hours (1 read repeat/minute), (4) 7.50 to 8 hours (1 read repeat/minute), and (5) 23.55 to 24.05 hours (1 read repeat/minute). Between reads, cells were kept in an incubator to promote CO_2_ intake and humidity equilibration. All baseline and final reads were made in a preheated Enspire multiplate reader at 37°C.

### Statistical analysis

Data were exported into Microsoft Excel 2013 (Redmond, WA). Background DMR (i.e., cell responses to assay buffer) were subtracted from all datasets, as described previously [21]. DMR data (n = 6 replicates/each condition) were expressed as mean ± standard error of the mean (SEM) and were plotted in a 2-dimensional (2D) dispersion graph. All graphs were generated using Graph Pad Prism 6 software (La Jolla, CA). Hyperbolic 1-phase decay graphs were evaluated using the curve-fitting function on Graph Pad. The plateau and span of the hyperbolic curve were expressed as mean ± standard deviation (SD).

Assay validation (robustness) was assessed by a Z’-factor calculation, in which c+ is the positive cytotoxic control (0.1% SDS), and c- is the negative nontoxic control (0.2% DMSO):

$$Z^{\prime }=1-\frac{3(\sigma_{c^{+}}+\sigma_{c^{-}})}{\left|\mu_{c^{+}}-\mu_{c^{-}}\right|}$$

A z’ ≥ 0.5 was regarded as an excellent (i.e., highly robust) assay. Cellular responses to the compounds were evaluated for significant differences at each time point by means of 1-way ANOVA (Excel 2013). The effects of compound type and incubation time on the cell response were evaluated using 2-way ANOVA (Excel 2013). Statistical significance was defined as p < 0.05.

## Results and discussion

**Fig 2:**
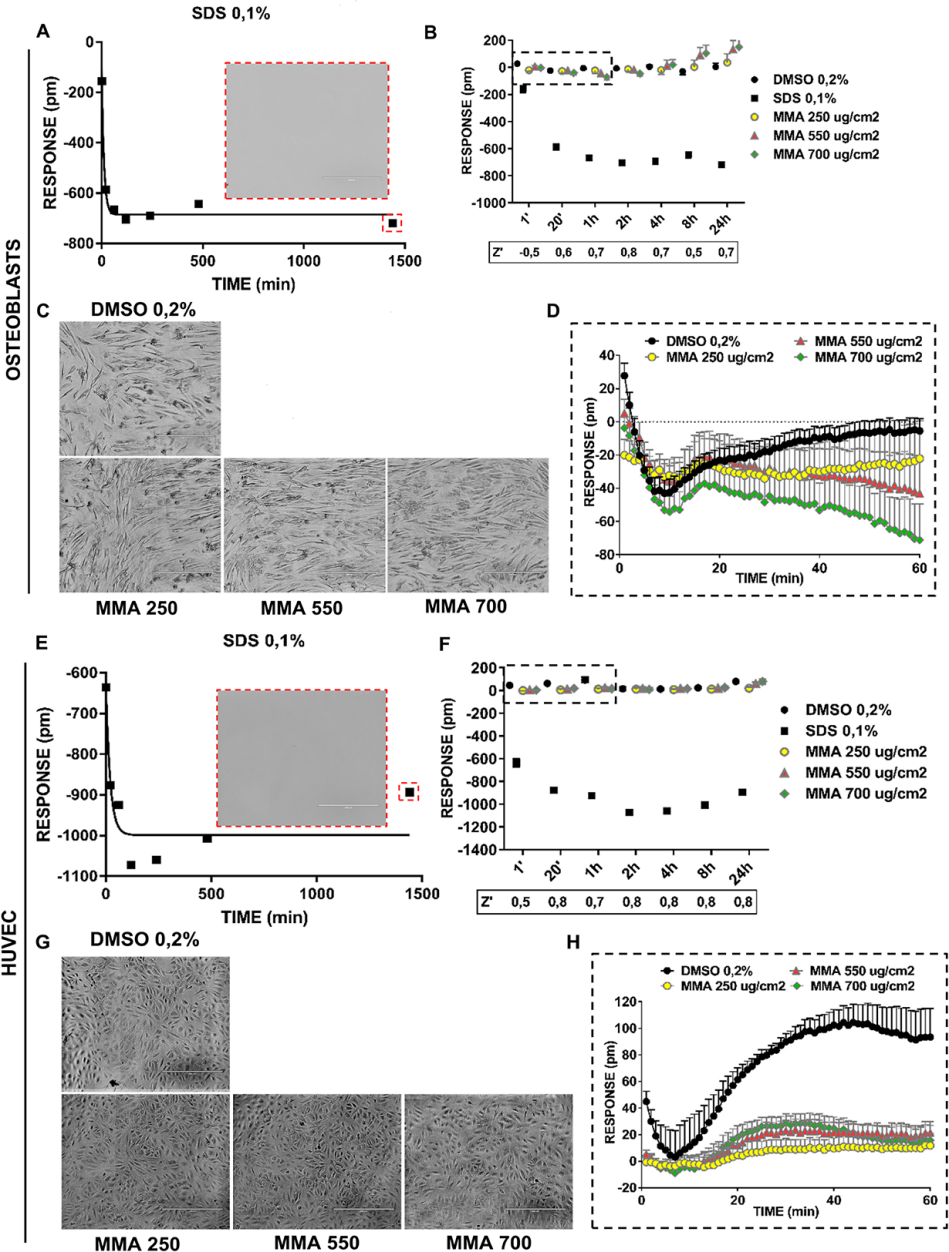
Effects of MMA on osteoblasts and HUVECs. A label-free assay (Epic, Corning) of cell response was carried out using 0.2% DMSO as the negative control and 0.1% SDS as the positive control. Osteoblasts and HUVECs were exposed to 3 concentrations of MMA: 250 μg/cm^2^, 550 μg/cm^2^, and 700 μg/cm^2^. Fig **2A–2D**: Results of the Epic assay on osteoblasts. Fig **2A**: The phenotypic profile of osteoblasts exposed to 0.1% SDS resembles a model of exponential decay and was specified as a hyperbolic 1-phase decay curve in GraphPad software. The red inset displays features of the osteoblasts 24 hours after SDS administration. Note the complete detachment of cells from the plastic. Fig **2B:** Multiple time point measurement findings were plotted in a 2D dispersion graph, with relative z’-factor analysis (n = 6). MMA-treated cells exhibited a range of responses comparable to those of DMSO-treated cells. Hence, the monomer was nontoxic to these cells. Fig **2C**: Representative photomicrographs of osteoblasts treated with 0.2% DMSO or with MMA at 1 of the 3 specified concentrations. After 24 hours, both DMSO-treated and MMA-treated samples displayed normal cell morphologies, indicating a lack of toxicity. Fig **2D**: Phenotypic profile analysis of osteoblasts treated with DMSO or MMA. Within 20 minutes, MMA-treated osteoblasts displayed a response comparable to those treated with DMSO. At 60 minutes, the responses to MMA differed in a dose-dependent manner (1-way ANOVA; p < 0.05). Fig **2E–2H**: Results of the Epic assay in HUVECs (endothelial cells). Fig **2E**: The phenotypic profile of HUVECs exposed to 0.1% SDS resembled a model of exponential decay and was specified as a hyperbolic 1-phase decay curve by GraphPad software. The red inset depicts features of the HUVECs 24 hours after SDS administration. Note the complete lack of plastic-adhering cells. Fig **2F:** Multiple time point measurement results were plotted in a 2D dispersion graph, with relative z’-factor analysis (n = 6). MMA-treated cells showed a range of responses comparable to those of DMSO-treated cells. Therefore, the MMA monomer is nontoxic in these cells. Fig **2G**: Representative photomicrographs of HUVECs treated with 0.2% DMSO or MMA at 1 of the 3 specified concentrations. After 24 hours, both DMSO-treated and MMA-treated samples displayed normal cell morphologies, indicating a lack of toxicity. Fig **2H**: Phenotypic profile analysis of HUVECs treated with DMSO or MMA. For all MMA concentrations tested, HUVEC responses were comparable and differed statistically from those of DMSO-treated cells at 20- and 60-minute time points (1-way ANOVA; p < 0.05).

Fig 2 depicts Epic assay findings involving 2 mesenchymal-derived cell types: osteoblasts and human umbilical vein endothelial cells (HUVECs). Cells exposed to sodium dodecyl sulfate (SDS)—the positive cytotoxic control—exhibited a progressive reduction in response, relative to the basal level for each cell type (−0.2 ± 2.1 pm and 1.7 ± 1.1 pm for osteoblasts and HUVECs, respectively; baseline data not shown). The response of these cells to SDS adhered to a model of exponential decay (Fig 2A and 2E). Specifically, the raw data was fitted to a hyperbolic 1-phase decay curve with a plateau of −685.3 ± 13.5 pm and −998.7 ± 37.2 pm for osteoblasts and HUVECs, respectively. This curve was associated with a span of 579.4 ± 36.9 pm and 376.1 ± 90.1 pm for osteoblasts and HUVECs, respectively (Fig 2A and 2E), suggesting a greater sensitivity of osteoblasts to MMA. This trend was associated with complete cell death in both cell lines, as confirmed by massive cell detachment at the 24-hour endpoint (Fig 2A and 2E; red inset).

Dimethyl sulfoxide (DMSO)-treated cells had negligible cytotoxicity for all time points evaluated (Fig 2B and 2F). In both osteoblasts and HUVECs, 1 hour after exposure to the compound, the Epic assay results enabled discrimination between a DMSO-like response (nontoxic) and an SDS-like response (toxic). Assay results yielded a z’-factor that indicated when assay validation was excellent (i.e., highly robust), based on a pre-established cut off of z’≥0.5; Fig 2B and 2F [22]. Findings of multipoint time analyses revealed that the assay was optimized within 1 hour from the administration of the stimulating compound. Thus, unlike the MTT assay, the Epic assay is not limited by a long incubation and a single read-out time.

At all time points, MMA-treated osteoblasts and HUVECs showed a range of responses that was comparable to those of cells treated with DMSO (i.e., DMSO-like profile; S1 Fig 2B, S2 Fig 2B, S3 Fig 2F and S4 Fig 2F). These findings were confirmed by microscopy results after 24 hours of incubation: both DMSO-treated and MMA-treated samples displayed a cell morphology indicative of a lack of toxic effect (Fig 2C and 2G). At 24 hours, the responses of osteoblasts to MMA treatment varied in a dose-dependent manner that was small but statistically significant (Fig 2B; 2-way analysis of variance [ANOVA]; Table 1A). This supported the hypothesis that Epic label-free technology was sufficiently sensitive to detect a biological effect in human primary cells. Statistically significant differences in cellular responses among incubation times also were found for osteoblasts and HUVECs treated with 0.2% DMSO or with MMA at 250, 550, or 700 μg/cm^2^ (p < 0.05; Table 1A). These data indicate a cellular reaction that may not necessarily be related to cytotoxicity but to a biological effect that should be further explored with multimodal approaches.

**Table 1:** Effects of compound type and incubation time on cellular responses.

***A***		
***OSTEOBLAST***		
***SOURCE***	**F-value**	**p-Value**
*INCUBATION TYPE + COMPOUND TYPE*	3,3	4,2 x 10^−5^
*INCUBATION TYPE*	14,1	1,1 x 10^−12^
*COMPOUND TYPE*	3,3	2 x 10^−2^
***B***		
***HUVEC***		
***SOURCE***	**F-value**	**p-Value**
*INCUBATION TYPE + COMPOUND TYPE*	2,1	9 x 10^−3^
*INCUBATION TYPE*	8,9	2,7 x 10^−8^
*COMPOUND TYPE*	13,1	1,4 x 10^−7^
***C***		
***NEURON***		
***SOURCE***	**F-value**	**p-Value**
*INCUBATION TYPE + COMPOUND TYPE*	1,8	3 x 10^−2^
*INCUBATION TYPE*	15,1	2,6 x 10^−13^
*COMPOUND TYPE*	70,5	7,5 x 10^-28^

**A-C**: Two-way ANOVA was applied to data analyses for all cell types. Compounds and incubation times, as well as the interactions of these factors, influence the responses of osteoblasts, HUVECs, and iPS-derived neurons.

We next performed a detailed examination of the responses to DMSO (negative control) and MMA (test compound), as recorded in raw form for osteoblasts and HUVECs by continuous-mode reading within 1 hour of compound administration (Fig 2D and 2H). DMSO-treated cells showed a cell type–dependent phenotypic profile. Osteoblasts displayed a profile involving an early N-DMR curve followed by restoration of the response to basal levels (Fig 2D). Osteoblasts treated with MMA had a response comparable to those treated with DMSO within the first 20 minutes. At 60 minutes post administration, the responses of osteoblasts to MMA differed in a dose-dependent manner (1-way ANOVA; p = 8.1 × 10^−5^). However, HUVECs treated with DMSO had an early N-DMR peak following by a P-DMR curve (Fig 2H). HUVECs treated with MMA at any of the 3 tested concentrations exhibited comparable responses that statistically differed from cells treated with DMSO at 20 and 60 minutes (1-way ANOVA; p = 6 × 10^−4^ and p = 3 × 10^−3^ for 20 minutes and 60 minutes, respectively). These findings indicate that osteoblasts and HUVECs have a DMSO-like cell profile when exposed to MMA. Thus, the monomer should be regarded as nontoxic at the tested concentrations (Fig 2D and 2H).

**Fig 3:**
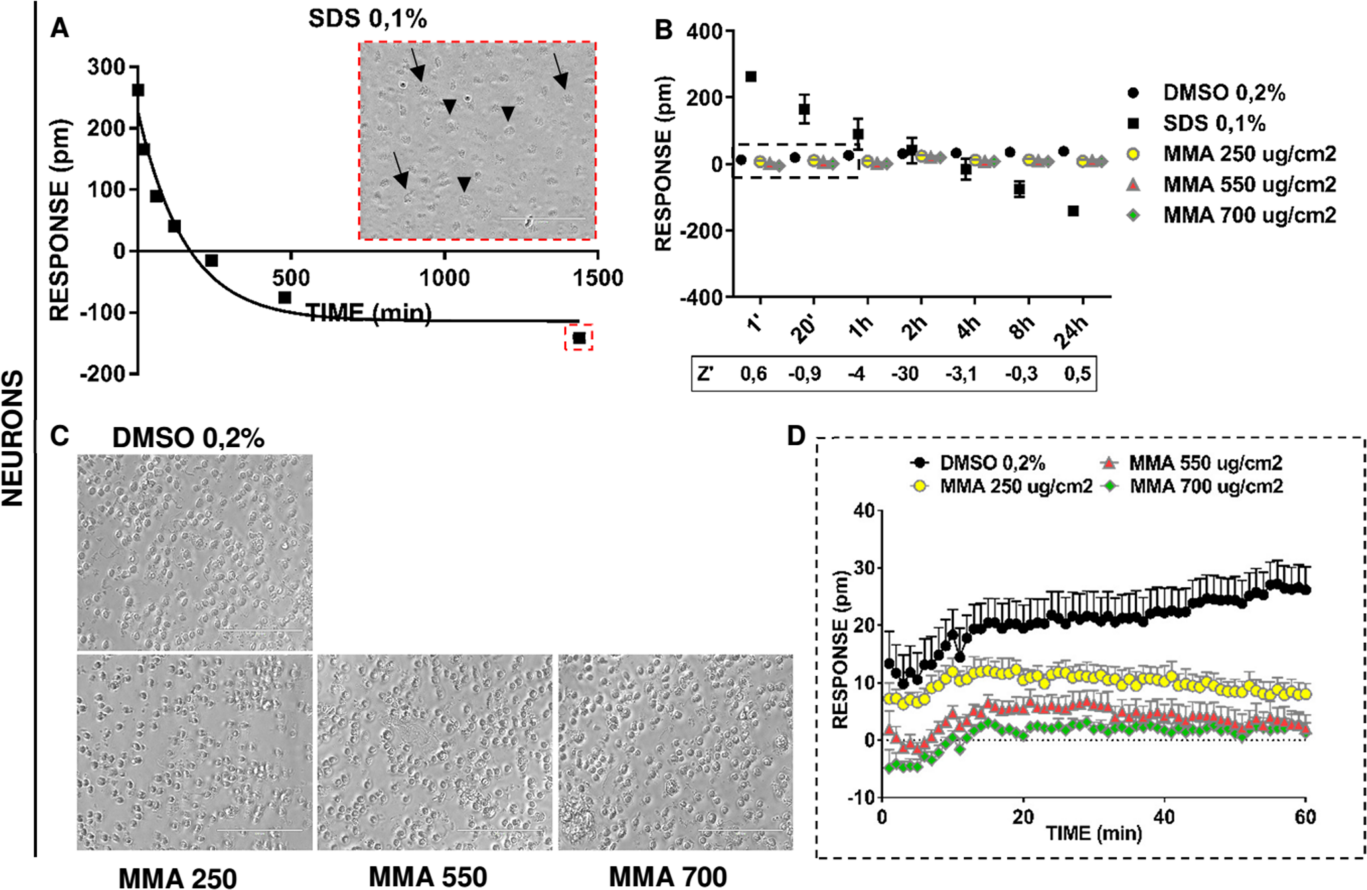
Effects of MMA on iPS-derived neurons, ascertained by the Epic label-free assay. The Epic label-free assay was carried out on iPS-derived neurons, with 0.2% DMSO and 0.1% SDS as negative and positive controls, respectively. Neurons were exposed to 1 of 3 concentrations of MMA: 250 μg/cm^2^, 550 μg/cm^2^, or 700 μg/cm^2^ of the cell layer surface. Fig **3A**: The phenotypic profile of iPS-derived neurons exposed to 0.1% SDS resembled a model of exponential decay. Within 1 minute after SDS addition, iPS-derived neurons responded with an early P-DMR. The red inset indicates features of the iPS-derived neurons 24 hours after SDS administration. Note the increase in cell volume (indicated by arrowheads) and the appearance of ruffling cell membranes (indicated by arrows). Fig **3B**: Multiple time point measurement findings were plotted in a 2D dispersion graph, with relative z’-factor analysis (n = 6). The early P-DMR for SDS-treated neurons was associated with a robust z’ factor (z ≥ 0.5) at 1 minute and after 24 hours. MMA-exposed cells exhibited a range of responses that was comparable to those administered DMSO. Hence, MMA was nontoxic to these cells at the tested concentrations. Fig **3C**: Representative photomicrographs of cells treated with 0.2% DMSO or MMA. After 24 hours, DMSO-treated and MMA-treated samples exhibited normal morphologic characteristics, which indicated a lack of toxicity. Fig **3D**: Phenotypic profile analysis of iPS-derived neurons treated with DMSO or MMA. At 60 minutes, iPS-derived neurons treated with prespecified concentrations of MMA exhibited responses that were comparable to each other and that differed significantly from those of DMSO-treated cells (1-way ANOVA; p < 0.05).

We next applied the Epic assay to explore the effects of MMA on an ectodermal cell type: iPS-derived neurons (Fig 3). These neurons displayed a very early positive response starting from 1 minute after compound addition (262.6 ± 9.6 pm), relative to the baseline response of 0.3 ± 0.4 pm (data not shown). From 1 minute to 24 hours, iPS-derived neurons showed a progressive reduction in response that adhered to a model of exponential decay (Fig 3A). The raw data were fitted to a hyperbolic 1-phase decay curve with a plateau of −113.8 ± 27.7 pm associated with a span of 343 pm (Fig 3A). This trend, contrary from that of osteoblasts and HUVECs, was not associated with complete cell detachment. Instead, iPS-derived neurons exhibited an increase in cell volume and deterioration of the cell membrane (Fig 3A, red inset). Conversely, DMSO-treated control neurons displayed negligible cytotoxicity for all time points (Fig 3B).

The iPS-derived neurons treated with prespecified concentrations of MMA showed a range of responses comparable with that of cells treated with DMSO (i.e., DMSO-like profile; S5 Fig 3B and S6 Fig 3B). These findings were confirmed after 24 hours by microscopic observation (Fig 3C). Both DMSO-treated and MMA-treated samples had cell morphologic findings indicative of a lack of toxicity (Fig 3C). Nevertheless, the 2-way ANOVA results showed a significant difference among cells that were exposed to 0.2% DMSO or to 250, 550, or 700 μg/cm^2^ of MMA (p < 0.05; Table 1C). As with results of osteoblasts and HUVECs exposed to MMA, these data do not necessarily indicate a toxic effect of MMA, but rather uncharacterized biological effects.

We then undertook an analysis of continuous-mode readings of the raw responses of iPS-derived neurons exposed to DMSO or MMA during the first 1 hour of the assay (Fig 3D). DMSO-treated neurons exhibited a unique phenotypic profile, with an early and low N-DMR curve, followed by restoration of the response to basal levels (Fig 3D). All MMA-treated iPS-derived neurons showed comparable responses that differed statistically from those of DMSO-treated cells at 1 hour (1-way ANOVA; p = 4.8 × 10^−6^). This finding confirms that the monomer is nontoxic to neuronal cells (Fig 3D).

Corning’s Epic label-free technology may be suitable for a broad range of applications, including drug discovery, pharmacogenomics, cell receptor signaling, cell migration, and viral titration [11,23,24,25]. To our knowledge, this study is the first to address the application of the Epic assay to biocompatibility testing of the MMA monomer on human osteoblasts, human endothelial cells, and iPS-derived neurons. Our data demonstrate that the MMA monomer, at the tested concentrations, is nontoxic to primary and iPS-derived human cells. These findings support the in vitro biocompatibility of MMA as a material for cranioplasty implantation [26].

The MTT assay requires a long incubation time, which precludes observation of the early-stage biological response. In contrast, Epic label-free technology generates results immediately after contact between cells and the stimulant. The Epic assay allows the investigator to rapidly evaluate the impact of a compound on specific cell types. Epic technology is sufficiently sensitive to detect subcytotoxic biological events that would not be detectable by microscopy or standard cytotoxicity assays. By enabling detection of the earliest hallmarks of a specific cellular response to a molecule, the Epic assay can help to focus and facilitate follow-up studies involving mRNA or miRNA expression profiling, electron microscopy, or proteomics. A combination of an initial Epic screen and these other modalities could minimize issues of oversensitivity associated with the Epic assay and could open a new field of the very early signs of cellular response to specific agents.

## Conclusion

In our study, we propose a cellular model using three different tissue-specified cell types in a label free manner (bone derived cells, endothelial derived cells and neuron-like cells). We applied the Epic Label Free technology in the field of biocompatibility of medical devices: it is able to discriminate the toxic nature of the SDS compared to the nontoxic DMSO compound. After the definition of the cell response to proper controls, we were able to evaluate the cellular response to MMA by comparing its profile within controls.

Our results support the further development of Epic label-free technology for biocompatibility studies, in which unlabeled, tissue-specific human cells may be challenged with medical devices in development for validation of the intended use and to obtain more comprehensive in vitro data on safety, as required by the new medical device regulation [27].

## Supporting information

S1 Fig Effects of MMA on osteoblasts and HUVECs. (B) Raw data of DMSO treated osteoblast. Multiple time point measurement findings were plotted in a 2D dispersion graph (n = 6).
(CSV)

S2 Fig Effects of MMA on osteoblasts and HUVECs. (B) Raw data of SDS treated osteoblast. Multiple time point measurement findings were plotted in a 2D dispersion graph (n = 6).
(CSV)

S3 Fig Effects of MMA on osteoblasts and HUVECs. (F) Raw data of DMSO treated HUVEC. Multiple time point measurement findings were plotted in a 2D dispersion graph (n = 6).
(CSV)

S4 Fig Effects of MMA on osteoblasts and HUVECs. (F) Raw data of SDS treated HUVEC. Multiple time point measurement findings were plotted in a 2D dispersion graph (n = 6).
(CSV)

S5 Fig Effects of MMA on iPS-derived neurons, ascertained by the Epic label-free assay. (B) Raw data of DMSO treated neurons. Multiple time point measurement findings were plotted in a 2D dispersion graph (n = 6).
(CSV)

S6 Fig Effects of MMA on iPS-derived neurons, ascertained by the Epic label-free assay. (B) Raw data of SDS treated neurons. Multiple time point measurement findings were plotted in a 2D dispersion graph (n = 6).
(CSV)
